# Detailed numerical evaluation of diffusion convection equation in layered reservoirs during tracer injection

**DOI:** 10.1038/s41598-023-40934-8

**Published:** 2023-09-11

**Authors:** Mahmood Moayyedi, Mohammad Sharifi, Mahdi Abbasi, Mahdi Shabani

**Affiliations:** Department of Petroleum Engineering, Amirkabir University of Technology, Tehran, Iran

**Keywords:** Petrology, Sedimentology, Chemical engineering

## Abstract

Characterization of heterogeneous reservoirs such as multilayered or fractured systems is an important issue in different disciplines such as hydrology, petroleum and geothermal systems. One of the popular methods that can be used for this purpose is tracer tests. Better understanding of the mechanisms of mass transfer (convection–diffusion process) is essential for having a proper test interpretation. In this study, the solutions of different scenarios of tracer flow in a pair of high and low-permeable layered reservoirs including convection and diffusion mechanisms are discussed. Although analytical solutions generally provided exact solutions, they involve several assumptions and might be hard to use for complex problems. As a result, numerical methods are selected for the investigation of different scenarios and addressing cases that are beyond access of analytical methods. In this study, several scenarios of considering diffusion and convection in low and high permeable zones and effective parameters on tracer concentration are investigated. According to the results of this study, the higher the porosity ratio of low to high permeable layer, the more time is needed to get the final concentration value. Also, by increasing the value of the dispersivity coefficient, the time needed to increase the concentration decreases. In other words, the sharp increase in concentration for lower times is seen in higher dispersivity values. The concentration profile variation is affected by Peclet number. The difference among concentration profiles in different cases is considerable, especially in low Peclet numbers where the diffusion mechanism is dominant. This behavior is more common in low permeable mediums such as multilayered tight or shale reservoirs.

## Introduction

Essentially reservoir heterogeneity is a variation in reservoir properties as a function of space and this challengeable factor exists in a lot of underground reservoirs. Therefore, identifying reservoir properties and also the mechanisms of fluid flow in heterogeneous systems is a vital goal of reservoir engineers for better characterization and reservoir management^1^. Any prediction of reservoir performance in these types of reservoirs based on limited information may not be true in some cases and should only serve as a starting point for analyzing the mediums. Multilayered and fractured reservoirs are two common types of heterogeneous systems. In these reservoirs, the fluid flow will be done in two or more different environments. Fractured reservoirs are composed of two different medium, including a matrix system with high porosity and low permeability, as well as fracture network with low porosity and high permeability in which reservoir fluid may be flow between these two mediums. On the other hand multi-layer reservoirs can also include different environments with porosity and permeability distribution, where their fluid flow calculation may be somewhat similar to fractured reservoirs. Therefore, a better understanding of the mechanism of mass transfer (convection–diffusion process) in these reservoirs and also the identification of affected parameters to flow is an important topic in fluids flow phenomena. Besides the different methods of reservoir characterization, the tracer flow tests are the methods for the better investigation of the mass transfer process in heterogeneous reservoirs. Different investigations have been done on tracer flow in heterogeneous reservoirs such as naturally fractured and multilayer reservoirs^2–7^.

To better evaluation of multilayered reservoirs as a type of heterogeneous system, Brigham and Smith proposed a tracer flow model in a water flooding process. This model was able to describe permeability heterogeneity in layered reservoirs^8^. Yuen et al. presented a computer algorithm to determine the degree of heterogeneity in these reservoirs using Brigham equations^9^. Abbaszadeh-Dehghani and Brigham (1984) reported analytical expressions to analyze tracer flow in layered reservoirs using a nonlinear optimization technique^10^. Sato and Abbaszadeh used tracer pulse test response as a function of layer number to evaluate the maximum produced concentration from homogeneous and multilayered reservoirs^11^. Sometimes later Samaniego et al. presented new models to evaluate tracer flow behavior in heterogeneous reservoirs using dimensionless parameters^12^. Shook used a flow-storage capacity diagram to get an idea of flow geometry, swept volume, and saturation distributions in the layered reservoir^13^.

Suarsana et al. used the comparison of tracer test results and analysis of connectivity injector and producer to investigate pilot water flooding performance in a multilayered reservoir^14^. Jason et al. show that changes in tracer concentration gradients can be good indicators of changes in porosity (or water saturation) between layers in multilayered reservoirs^15^. Shen et al. presented a new formulation to evaluate multilayered reservoir behavior regarding limited crossflow^16^. Bahamon and Mora described a method to evaluate water channeling and assess sweep efficiency improvements in a multilayered high water cut field using an inter-well tracer test to better perform the water flooding process^17^. Davidescu et al. used a tracer test in the polymer and water injection process to evaluate the sweep efficiency of a multilayered reservoir^18^.

Some investigations also worked on tracer flow in the matrix-fracture mediums in fractured reservoirs as other types of heterogeneous systems. In 1986 a mathematical model was developed to estimate fracture width using tracer test results. The results showed good agreement in comparison with laboratory data^19^. After that Raven et al. investigated the tracer flow in a fractured geothermal reservoir by introducing the Peclet dimensionless number to evaluate the time of tracer breakthrough in matrix fracture media^20^. Ramirez et al. investigated the response of the tracer test in matrix fracture medium and tried to estimate fracture opening and also matrix diffusion coefficient in different geometries^7,21^. Samaniego and Rodriguez used the tracer data in an observation well to estimate some reservoir parameters in a fractured reservoir using a trial method^22^. Qasem et al. showed that parameters such as porosity and permeability and their relationship significantly affect tracer results in fracture-matrix systems^23^. After that Samaniego et al. in 2005 presented a model to investigate the tracer flow in the homogenous and heterogeneous reservoir. They reported that some reservoir parameters such as block size and also dispersion coefficient can be estimated using tracer analysis^12^. Kocabas and Maier used analytical and numerical models to investigate the tracer flow in a fractured reservoir^24^. Haddad et al. used analytical methods to estimate the dispersion coefficient in different geometries of matrix-fracture media by solving the convection–diffusion equation^25^. Aymen et al. used the tracer response in analytical and numerical models to estimate water cut in fractured reservoirs^26^. Abbasi et al. used tracer data and discussed the analytical solution of the mass transfer equation to evaluate the shape factor in a fractured reservoir^27^. Jing et al. estimated the future performance of an ultralow-permeability fractured reservoir using tracer test and production data^28^. Kumar et al. reported that the combination of tracer data, completion data and also stimulation data in a machine-learning framework could show better dynamic behavior in a fracture network system^29^. Also a brief history of activities performed in the analysis of tracers is presented in Appendix C^11,12,16,18,24,30–57^.

In this study, first, an introduction to the general convection–diffusion equation is presented. Then, tracer flow in a two-layered system as a heterogeneous reservoir is evaluated in three scenarios. In the first scenario (Base Case), convection in higher permeable and diffusion from higher to lower permeable layers are dominant mechanisms. In the second case, the diffusion toward the lower permeable layer (L2) is also included in the evaluation. Finally, the convection mechanism toward L2 is added to the calculations. In this work, three different cases of mass transfer in tracer injection with different boundary conditions are investigated. Because only the analytical solution of the simplest case is available in the literature, the numerical methods are considered to solve mass transfer equations in these cases. Accordingly in the next section the results of analytical and numerical methods are compared for base simple case to check the validity of numerical methods. After that, the sensitivity analysis of affecting parameters in the convection–diffusion equation is done. Finally, a comparison among different cases of tracer flow in these layers is investigated.

## Methodology

### General convection–dispersion equation

According to physical phenomena, a solute (such as tracer) molecular diffusion occurs from higher to lower concentration. Diffusion in porous media can be explained using Fick's first law. The mass of diffused fluid is related to the concentration gradient as below:

1 $${\text{F}} = - {\text{D}}_{{\text{d}}} \left( {\frac{dC}{{dx}}} \right)$$

in which F, D_d_ and ($\frac{dC}{{dx}}$) are mass flux, diffusion coefficient and concentration gradient, respectively. On the other hand, if concentration changes with time, Fick’s second law is used to show the diffusion variation as a function of space and time. This equation in one dimension may be expressed as:

2 $$\frac{\partial C}{{\partial t}} = D_{d} \frac{{\partial^{2} C}}{{\partial z^{2} }}$$

Besides the diffusion phenomena, convection is another important mechanism for solute transport in porous media. The one-dimensional advection transport equation is in the following form where *v*_x_ is the average linear velocity.

3 $$\frac{\partial C}{{\partial t}} = - v_{x} \frac{\partial C}{{\partial x}}$$

According to studies done by Ogata and Bear, the convection–dispersion equations in representative elemental volume (REV) of a porous medium are in the following forms^58,59^:

4 $${\text{advective}}\,{\text{transport}} = {\text{v}}_{i} n_{e} CdA$$

5 $${\text{Dispersive}}\,{\text{transport}} = n_{e} D_{i} \frac{\partial C}{{\partial i}}dA$$

where n_e_, *dA*, and *i* are effective porosity, the cross-sectional area of the element, and the normal direction to cross-section, respectively. The total mass of solute per unit cross-sectional area transported in the *i* direction per unit time, Fi, is the sum of the advective and the dispersive transports are given by:

6 $${\text{F}}_{i} = {\text{v}}_{i} n_{e} C - n_{e} D_{i} \frac{\partial C}{{\partial i}}$$

The amount of solute entering and leaving the representative elemental volume is:

7 $${\text{F}}_{x} dzdy + F_{y} dzdx + F_{z} dxdy\quad \quad {\text{(entering}}\,{\text{element)}}$$

8 $$\left( {{\text{F}}_{x} + \frac{{\partial {\text{F}}_{x} }}{\partial x}} \right)dzdy + \left( {F_{y} \frac{{\partial F_{y} }}{\partial y}} \right)dzdx + \left( {F_{z} \frac{{\partial F_{z} }}{\partial z}} \right)dxdy\quad \quad {\text{(leaving element)}}$$

The mass of solute variation in representative elemental volume also can be expressed as:

9 $$n_{e} \frac{\partial C}{{\partial t}}dxdydz$$

Using the mass conservation law, the rate of mass change in the representative elemental volume must be equal to the difference in the mass of the solute entering and leaving:

10 $$n_{e} \frac{\partial C}{{\partial t}} = - \left( {\frac{{\partial {\text{F}}_{x} }}{\partial x} + \frac{{\partial F_{y} }}{\partial y} + \frac{{\partial F_{z} }}{\partial z}} \right)$$

Substituting Eq. (6) into Eq. (10) and canceling ne from both sides yields the general 3D convection–diffusion equation in Cartesian coordinate:

11 $$\frac{\partial C}{{\partial t}} = \left[ {\frac{\partial }{\partial x}\left( {{\text{D}}_{x} \frac{\partial C}{{\partial x}}} \right) + \frac{\partial }{\partial y}\left( {{\text{D}}_{y} \frac{\partial C}{{\partial y}}} \right) + \frac{\partial }{\partial z}\left( {{\text{D}}_{z} \frac{\partial C}{{\partial z}}} \right)} \right] - \left[ {\frac{\partial }{\partial x}({\text{v}}_{x} C) + \frac{\partial }{\partial y}({\text{v}}_{y} C) + \frac{\partial }{\partial z}({\text{v}}_{z} C)} \right]$$

The above relation is a 3D mass transfer equation in porous media without any chemical reaction. In a homogeneous medium, D_x_, D_y_, and D_z_ do not vary in space. However, because the coefficient of the hydrodynamic dispersion is a function of the flow direction, even in an isotropic, homogeneous medium, then D_x_#D_y_#D_z_ should be considered in calculations.

If dilution of solute at the advancing edge of flow occurs, the mixing will be mechanical dispersion. In this condition, the mixing which occurs along the advancing edge of flow is longitudinal dispersion and the mixing in the direction normal to the flow path is called transverse dispersion.

If the mechanical dispersion is described by Fick’s law for diffusion and it is assumed as a function of the average linear velocity, then the coefficient of mechanical dispersion will be calculated by introducing the dispersivity. Dispersivity denoted by α is a property of porous media. Dispersivity times the average linear velocity i.e. α.v will be called the coefficients of mechanical dispersion The combination of this parameter and molecular diffusion (D*) is called hydrodynamic dispersion coefficient which is represented by the following formulas;

12 $$D_{L} = \alpha_{L} \nu_{i} + {\text{D}}^{*}$$

13 $$D_{T} = \alpha_{T} \nu_{i} + D^{*}$$

where *D*_*L*_ and *D*_*T*_ are hydrodynamic dispersion coefficient parallel (longitudinal) and perpendicular (transverse) to the principal direction of flow.

For those domains where the average linear velocity *v*_*i*_ is uniform in space, Eq. (11) for one-dimensional flow in a homogeneous isotropic media will be presented in the following form where only longitudinal dispersion coefficient (*D*_*L*_) will be used:

14 $$\frac{\partial C}{{\partial t}} = {\text{D}}_{L} \frac{{\partial^{2} C}}{{\partial i^{2} }} - {\text{v}}_{i} \frac{\partial C}{{\partial i}}$$

Also for two-dimensional flow with the direction of flow parallel to the x-axis, Eq. (11) will be presented in the following form using longitudinal and transverse dispersion coefficients:

15 $$\frac{\partial C}{{\partial t}} = \left( {{\text{D}}_{L} \frac{{\partial^{2} C}}{{\partial i^{2} }}} \right) + \left( {{\text{D}}_{T} \frac{{\partial^{2} C}}{{\partial j^{2} }}} \right) - {\text{v}}_{i} \frac{\partial C}{{\partial i}}$$

*D*_*L*_*—*the longitudinal hydrodynamic dispersion (L^2^/*T*); *D*_*T*_—the transverse hydrodynamic dispersion (L^2^/T).

On the other hand, the radial flow from a well in polar coordinate can be written as bellow^59^:

16 $$\frac{\partial C}{{\partial t}} = \frac{1}{r}\frac{\partial }{\partial r}\left( {rD\frac{\partial C}{{\partial r}}} \right) - \frac{\partial (VC)}{{\partial r}} = \frac{\partial }{\partial r}\left( {D\frac{\partial C}{{\partial r}}} \right) + \frac{D}{r}\left( {\frac{\partial C}{{\partial r}}} \right) - V\frac{\partial C}{{\partial r}} = D\frac{{\partial^{2} C}}{{\partial r^{2} }} + \frac{D}{r}\left( {\frac{\partial C}{{\partial r}}} \right) - V\frac{\partial C}{{\partial r}}$$

where r and V are the radial distance of the well and average pore velocity respectively.

In this study, the above equations will be discretized and solved numerically to investigate the convection–diffusion mechanisms in heterogeneous reservoirs. These types of reservoirs may be presented by high and low-permeable systems such as Matrix-Fracture or multilayered media.

### Numerical modeling of convection–diffusion flow in layered reservoirs

Numerical modeling is a powerful tool to evaluate physical phenomena such as convection diffusion in heterogeneous reservoirs. Although the analytic solution of an equation or expression is the exact solution and guarantee that the resulting quantitative predictions are accurate, due to some complicated boundary conditions and limitations of analytical methods, numerical calculations are more useful. In the following sections of this study, numerical methods are used to evaluate physical phenomena such as convection diffusion in heterogeneous reservoirs. This study investigates the convection–diffusion mechanism of two-layered reservoirs with different degrees of contrast between layer's permeability. Different cases of flow in this reservoir are introduced to solve by numerical modeling.

First of all, it is assumed that tracer flow is occurred in two-layered systems from the wellbore toward the higher permeable layer (L1) by convection mechanism. Also, diffusion in vertical direction is the dominant mechanism for mass transfer from the higher permeable layer to the lower permeable layer (L2). This case may be similar to a dual porosity system in fractured reservoirs. In this case (base case), diffusion exist in L1 but it is not the dominant mechanism.

In the second case, the diffusion toward the lower permeable layer (L2) is also included in the evaluation. Finally, the convection mechanism toward L2 will be added to the calculations.

To solve the convection–diffusion equation, first of all, the dominant mechanism in L2 will be assumed to be the diffusion from L1 in the vertical direction using the following formula where subscript numbers 1 and 2 represent the higher and permeable layer properties respectively:

17 $$\frac{{\partial C_{2} }}{\partial t} = {\text{D}}_{2} \frac{{\partial^{2} C_{2} }}{{\partial z^{2} }}$$

where D2 is the diffusion coefficient in L2. Also, the general convection–diffusion equation in L1 will be presented in the following form and is similar for all cases. The equation is in the one-dimensional system in radial coordinates. The terms on the right-hand side of the equation are velocity-dependent.

18 $$\frac{{\partial C_{1} }}{\partial t} + \frac{{\phi_{2} }}{{\phi_{1} }}\frac{{D_{2} }}{{h_{b/2} }}\frac{{\partial C_{2} }}{\partial Z}\left| {_{{\left| {Z = } \right.h_{b/2} }} } \right. = \frac{1}{r}\left[ {\left( {\left( {D_{1} r + \frac{{\alpha q_{o} }}{{2\pi h\phi_{1} }}} \right)\frac{{\partial^{2} C_{1} }}{{\partial r^{2} }}} \right)} \right] + \frac{1}{r}\left[ {\left( {D_{1} - \frac{{q_{o} }}{{2\pi h\phi_{1} }}} \right)\frac{{\partial C_{1} }}{\partial r}} \right]$$

In the above equation the terms $\frac{{\phi_{2} }}{{\phi_{1} }}$, *h*_*b*/2_, *α*, *q*_*o*_, *h* and *D*_1_ represent the porosity ratio of L_2_ to L_1_, thickness of layer L2, dispersivity, production rate, thickness of L1 and diffusion coefficient in L1 respectively. The derivation of this equation is presented in Appendix A.

If the diffusion coefficient term is assumed to equal zero in high permeability medium which is not unusual, then the equations will be presented in the following short form:

19 $$\frac{{\partial C_{1} }}{\partial t} + \frac{{\phi_{2} }}{{\phi_{1} }}\frac{{D_{2} }}{{h_{b/2} }}\frac{{\partial C_{2} }}{\partial Z}\left| {_{{\left| {Z = } \right.h_{b/2} }} } \right. = \frac{1}{r}\frac{{\alpha q_{o} }}{{2\pi h\phi_{1} }}\frac{{\partial^{2} C_{1} }}{{\partial r^{2} }} - \frac{{q_{o} }}{{2\pi rh\phi_{1} }}\frac{{\partial C_{1} }}{\partial r}$$

To better solve the tracer flow in porous media according to convection–diffusion equations, the following dimensionless parameters were introduced:

20 $$C_{D2} = \frac{{C_{i} - C_{2} }}{{C_{i} - C_{o} }}$$

21 $$C_{D1} = \frac{{C_{i} - C_{1} }}{{C_{i} - C_{o} }}$$

22 $$t_{D} = \frac{{D_{m} t}}{{r_{w}^{2} }}$$

23 $$h_{R} = \frac{{h_{b} }}{{r_{w} }}$$

24 $$Z_{D} = \frac{Z}{{r_{w} }}$$

25 $$D_{2\_1D} = \frac{{D_{1} }}{{D_{2} }}$$

26 $$\alpha_{D} = \frac{\alpha }{{r_{w} }}$$

27 $$r_{D} = \frac{r}{rw}$$

28 $$P_{e2\_1} = \frac{{\frac{{rq_{o} }}{{2\pi rh\phi_{1} }}}}{{D_{2} }} = \frac{{rV_{1} }}{{D_{2} }}$$

The terms, C_1D_, C_D2_, and t_D_ represent dimensionless concentrations of L1 and L2 layers and dimensionless time respectively. Also, h_R_ is the dimensionless ratio of L2 layer thickness to wellbore radius and Z_D_ represents the dimensionless ratio of the vertical coordinate of the L2 layer to wellbore radius. The terms α_D_ and D_2_1D_ show dimensionless dispersivity and dimensionless diffusion coefficients of L1 to L2 respectively. On the other hand, to define the relation between convection and diffusion mechanisms, dimensionless Peclet number is introduced. Peclet number (P_e_) is the ratio of advective velocity to the molecular diffusion coefficient. Fundamentally the value of this number is lower in regions of lower permeable than higher permeable layers.

**Figure 1:**
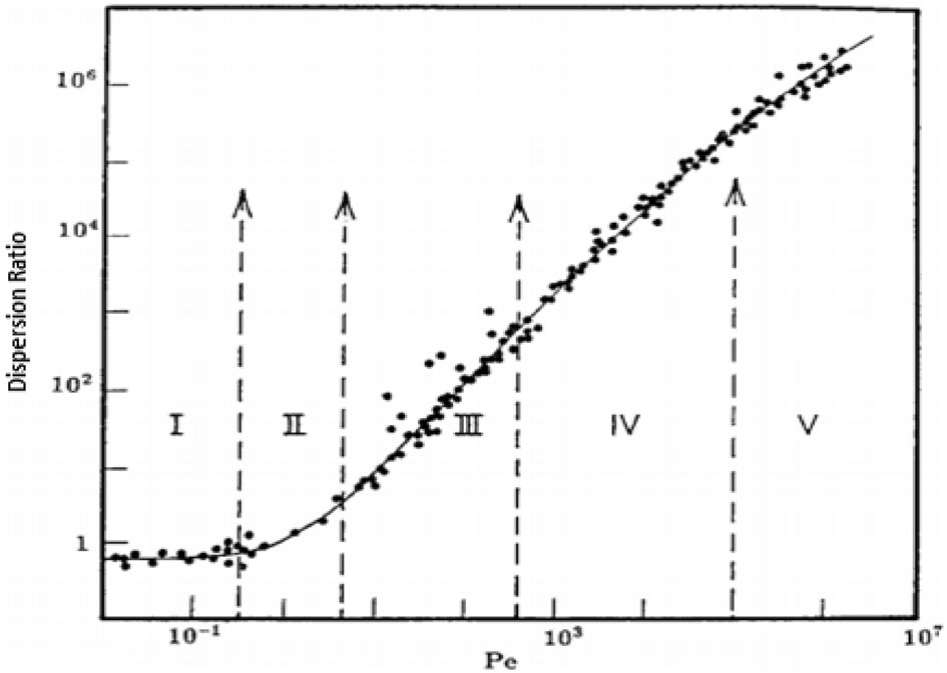
Dependence of the dispersion coefficient ratio on Peclet number Pe and the various dispersion regimes^60,61^.

According to the literature, five dispersion flow regimes can be described in different mediums. These regimes may be divided according to the value of the Peclet number and its dependency on the experimentally estimated dispersion ratio. For example for sand-pack medium, Fig. 1 shows the five dispersion regimes as follows:

1. Pe < 0.3 presents the diffusion regime in which convection is not the case and diffusion is dominant.
2. The regime 0.3 < Pe < 5 is the transition zone in which the effect of convection is important but the diffusion effect is strong.
3. In the range 5 < Pe < 300 the convection dominates dispersion, but the effect of molecular diffusion cannot be neglected.
4. When 300 < Pe < 10^5^ the flow regime is the purely convective regime.
5. For Pe > 10^5^ dispersion is in the turbulent regime in which the Peclet number is no longer the only correlating parameter, as the Reynolds number should also be considered. In this condition or flow through porous media, this regime is not of interest.

In this study, the relation between the velocity of the high permeable layer (L1) and diffusion between high and low permeable layers (L2) will be presented with Pe_2_1_ as defined in Eq. (28). On the other hand, the relation between convection and diffusion in the low permeable layer will be introduced with Pe_2_ in Eq. (46). According to Darcy law and assuming the same other properties except for permeability; the value of the Peclet number in L2 is lower than its value in L1 due to lower fluid velocity.

Using the predefined dimensionless parameters Eqs. (17), (18), and (19) will be presented in dimensionless form as Eqs. (29), (30), and (31) respectively:

29 $$\frac{{\partial c_{D2} }}{{\partial t_{D} }} = \frac{{\partial^{2} c_{D2} }}{{\partial z_{D}^{2} }}$$

30 $$\frac{{\partial C_{1D} }}{{\partial t_{D} }} = \alpha_{D} P_{e2\_1} \frac{1}{{r_{D} }}\frac{{\partial^{2} C_{1D} }}{{\partial r_{D}^{2} }} - \frac{1}{{r_{D} }}P_{e2\_1} \frac{{\partial C_{1D} }}{{\partial r_{D} }} - \frac{{\phi_{2} }}{{\phi_{L1} }}\frac{2}{{h_{r} }}\frac{{\partial C_{2D} }}{{\partial Z_{D} }}\left| {_{{\left| {Z_{D} = } \right.h_{r/2} }} } \right.$$

31 $$\begin{aligned} \frac{{\partial C_{1D} }}{{\partial t_{D} }} + \frac{{\phi_{2} }}{{\phi_{1} }}\frac{2}{{h_{R} }}\frac{{\partial C_{2D} }}{{\partial Z_{D} }}\left| {_{{\left| {Z_{D} = } \right.h_{R} /2}} } \right. & = \frac{1}{{r_{D} }}\left[ {(D_{2\_1D} - P_{e2\_1} )\frac{{\partial C_{1D} }}{{\partial r_{D} }}} \right] + \left( {D_{2\_1D} \frac{{\partial^{2} C_{1D} }}{{\partial r_{D}^{2} }}} \right) + \frac{1}{{r_{D} }}P_{e2\_1} \alpha_{D} \frac{{\partial^{2} C_{1D} }}{{\partial r_{D}^{2} }} \\ \to \frac{{\partial C_{1D} }}{{\partial t_{D} }} + \frac{{\phi_{2} }}{{\phi_{1} }}\frac{2}{{h_{R} }}\frac{{\partial C_{2D} }}{{\partial Z_{D} }}\left| {_{{\left| {Z_{D} = } \right.h_{R} /2}} } \right. & = \left\{ {\frac{1}{{r_{D} }}\left[ {(D_{2\_1D} - P_{e2\_1} )\frac{{\partial C_{1D} }}{{\partial r_{D} }}} \right]} \right\} + \left( {D_{2\_1D} + \frac{{P_{e2\_1} \alpha_{D} }}{{r_{D} }}} \right)\frac{{\partial^{2} C_{1D} }}{{\partial r_{D}^{2} }} \\ \end{aligned}$$

### Introduction to different cases of flow in numerical modeling

In this section, the convection–diffusion equation for two layered defined systems will be presented in three following cases named I, II, and III. In all cases, it is assumed that the convection from L1 to L2 is ignored. Also, the diffusion in L1 wouldn’t include in calculations due to its low effect on mass transfer. It is considered that Eq. (31) will be solved numerically as the mass transfer equation of L1 for all of the following cases. Table 1 describes introduced mass transfer mechanisms in defined cases.

**Table 1:** Description of mass transfer mechanisms in defined cases.

Cases name	Description
I	Convection in L1
	Diffusion from L1 to L2
II	Convection in L1
	Diffusion from L1 to L2
	Diffusion in L2
III	Convection in L1
	Convection in L2
	Diffusion from L1 to L2
	Diffusion in L2

#### Convection in L1—diffusion from L1 to L2

**Figure 2:**
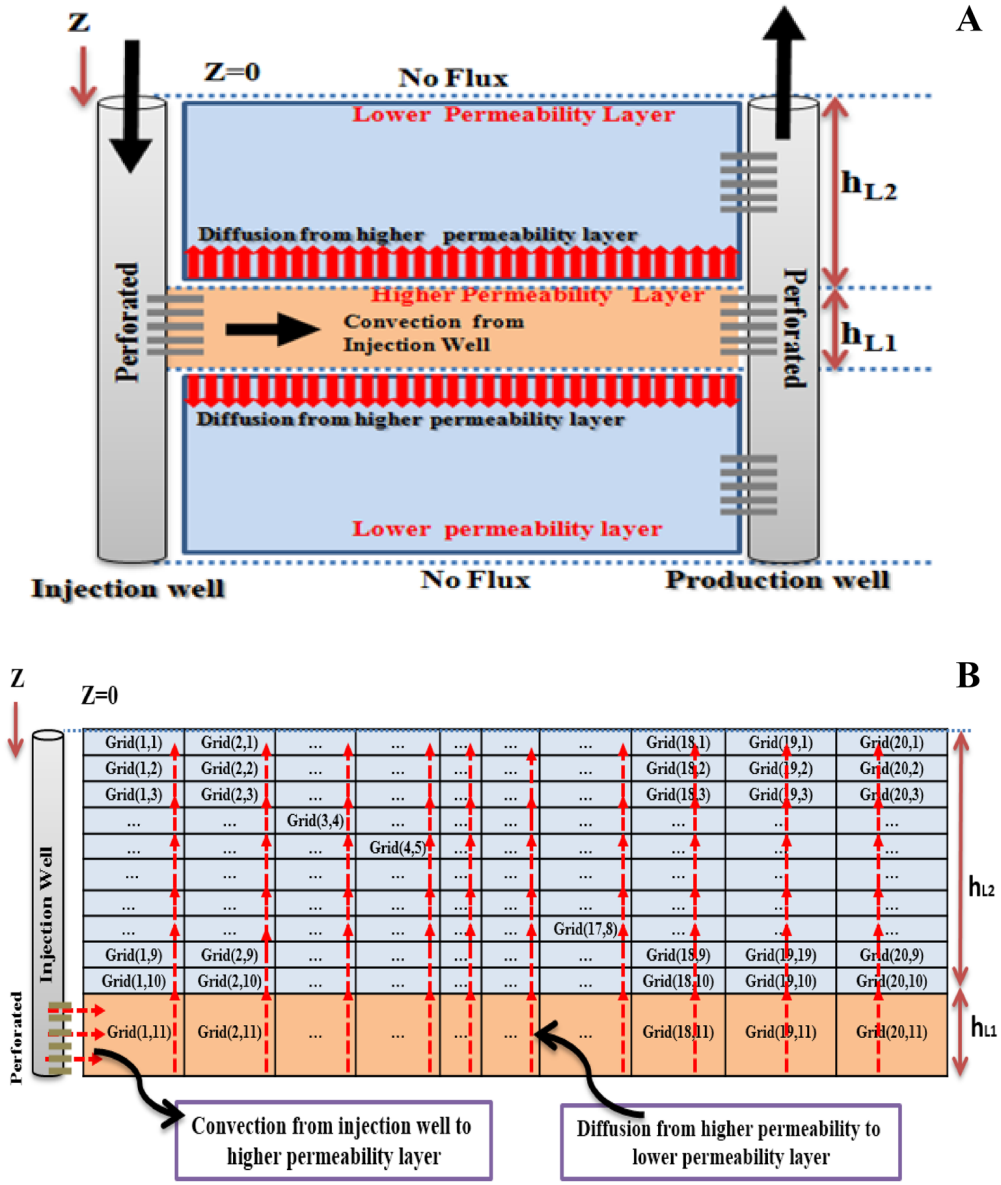
Schematic of case I (**A**)/Layer discretization for a sample reservoir N_L1_ = 20 and N_L2_ = 10. Total number of grids in L1 and L2 are 20 and 200 grids respectively (**B**).

The schematic of diffusion-convection flow in pours media, in this case, is shown in Fig. 2A where h_high_k_ and h_low_k_ are the half length of L1 and L2 layers respectively. In this figure, the convection exists in L1 and the diffusion is considered to exist form L1 to L2. As is presented in Fig. 2B after discretization and introduction of the coefficient and the known matrixes, the unknown matrix will be solved using numerical methods. The figure shows a sample of grids discretization in radial (r) and vertical (z) directions respectively. The number of total grids will be calculated using the following formula:

32 $$NN = \left( {N_{low\_k} + 1} \right)*( \, N_{high\_k} )$$

where N_low_k_ and N_high_k_ are the number of grids in vertical and radial directions for lower and the high permeable layers respectively. The total number of grids in high permeable layer equals N_high_k_ and the total number of grids in the low permeable layer equals NN-N_high_k_. It is assumed that L1 and L2 are divided by 10 grids for low permeable layer and 20 grids for high permeable layer in Fig. 2B and a total of 220 grids according to above formula. In this condition the location of each grid will be described by (r,z). It is assumed that the numerical calculations will be done for a pair of higher-lower permeability layers.

In this case, Eq. (30) will be used to solve diffusion from L1 to L2 in which the initial and boundary conditions are in the following forms:

33 $$C_{2D} (z_{D} ,t_{D} ) = 0\quad \quad t_{D} = 0\quad \quad 0 \le z_{D} \le h_{R} { / }2$$

34 $$C_{2D} \, \left( {z_{D} ,t_{D} } \right) = C_{1D} \quad \quad t_{D} > 0\quad \quad z_{D} = h_{R} { / }2$$

35 $$\partial C_{2D} \, \left( {z_{D} ,t_{D} } \right)/\left( {\partial z_{D} } \right) = 0\quad t_{D} > 0\quad \quad z_{D} = 0$$

Also to solve the mass transfer equation in L1 according to Eq. (28), the initial and boundary conditions will be presented in the following form:

36 $$C_{D1} \left( {z_{D} ,t_{D} \, } \right) = 0\quad \quad t_{D} = 0\quad \quad r_{wD} \le r_{D} \le \infty$$

37 $$C_{D1} \left( {z_{D} ,t_{D} \, } \right) = 1\quad t_{D} > 0\quad \quad r_{D} = 1$$

38 $$C_{D1} \left( {z_{D} ,t_{D} \, } \right) = 0\quad \quad t_{D} > 0\quad \quad r_{D} \to \infty$$

The diffusion term may be included in the mass transfer of the higher permeability layer (L1), but it is not so important because the convection is the dominant mechanism in this layer.

#### Convection in L1—diffusion from L1 to L2—diffusion in L2

**Figure 3:**
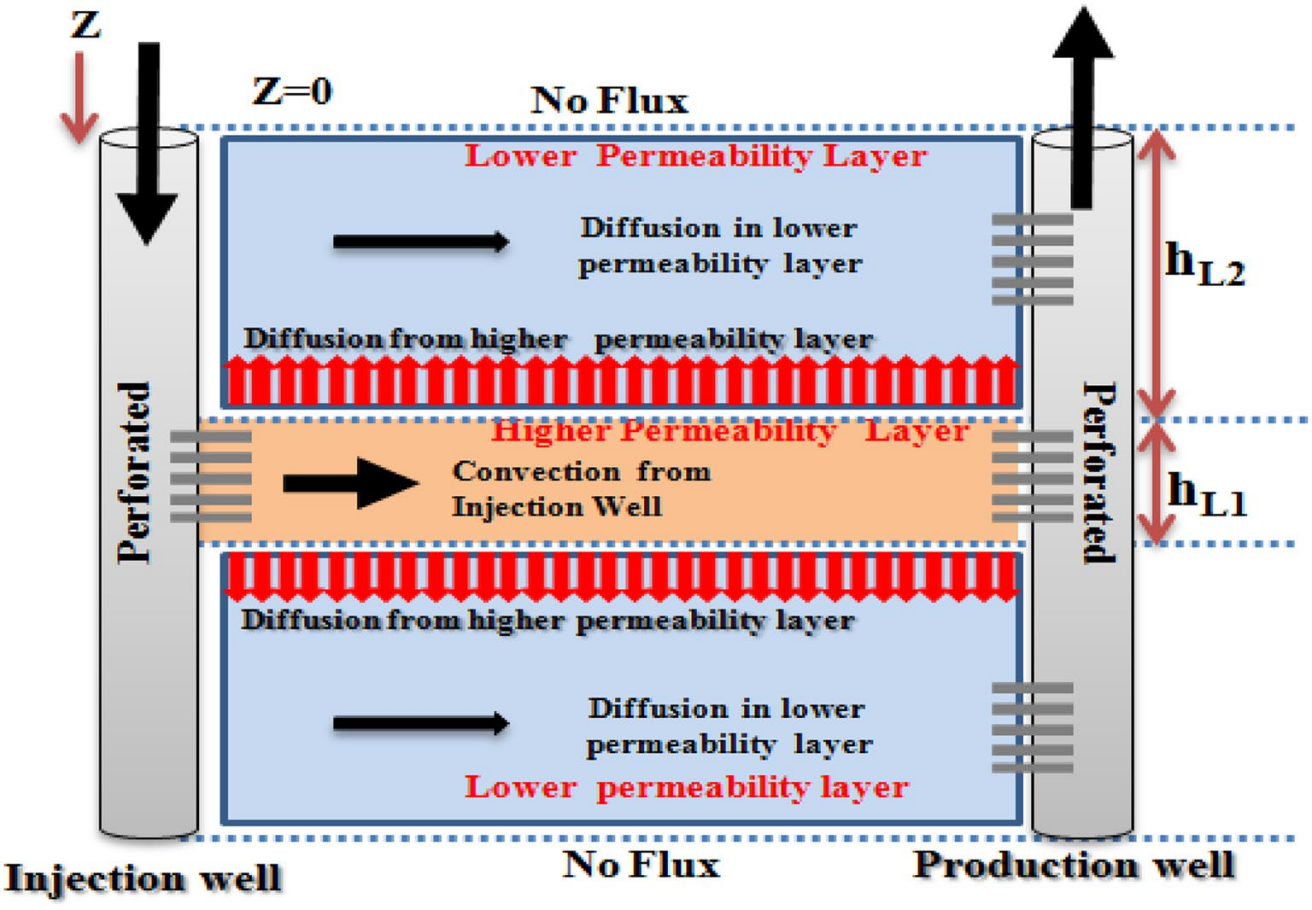
Schematic of convection–diffusion tracer mass transfer (convection in L1—diffusion from L1 to L2—diffusion in L2).

In this case, the diffusion toward the L2 is introduced as another existing mechanism of mass transfer in addition to diffusion from L1 to L2. The schematic of diffusion-convection flow, in this case, is shown in Fig. 3.

In this condition the mass transfer equation in L2 can be shown in the following forms:

39 $$\frac{{\partial C_{2} }}{\partial t} = D_{2} \frac{{\partial^{2} C_{2} }}{{\partial r^{2} }} + \frac{{D_{2} }}{r}\left( {\frac{{\partial C_{2} }}{\partial r}} \right) + D_{2} \frac{{\partial^{2} C_{2} }}{{\partial Z^{2} }}\quad \quad {\text{(dimensional)}}$$

40 $$\frac{{\partial C_{2D} }}{{\partial t_{D} }} = \frac{{\partial^{2} C_{2D} }}{{\partial r_{D}^{2} }} + \frac{1}{{r_{D} }}\frac{{\partial C_{2D} }}{{\partial r_{D} }} + \frac{{\partial^{2} C_{2D} }}{{\partial Z_{D}^{2} }}\quad \quad {\text{(dimensionless)}}$$

Also, the initial and boundary conditions (BC) in this case are presented below:

41 $$C_{2D} (z_{D} ,t_{D} ) = 0\quad \quad t_{D} = 0\quad \quad 0 \le z_{D} \le h_{R} { / }2$$

42 $$C_{2D} \, \left( {z_{D} ,t_{D} } \right) = C_{fD} \quad \quad t_{D} > 0\quad \quad z_{D} = h_{R} { / }2$$

43 $$\partial C_{2D} \, \left( {z_{D} ,t_{D} } \right)/\left( {\partial z_{D} } \right) = 0\quad \quad t_{D} > 0\quad \quad z_{D} = 0$$

44 $$C_{2D} \left( {r_{D} ,t_{D} \, } \right) = 1\quad \quad t_{D} > 0\quad \quad r_{D} = 1$$

45 $$C_{2D} \left( {r_{D} ,t_{D} \, } \right) = 0\quad \quad t_{D} > 0\quad \quad r_{D} \to \infty$$

In addition to boundary conditions in the previous case, two new B.C‘s (Eqs. 44 and 45) will be added to numerically solve the mass transfer equation. According to new B.C., the tracer concentration near the wellbore (injection point, r_D_ = 1) is equal to one and its value in far distances from the injection point will be zero.

#### Convection in L1—convection in L2—diffusion from L1 to L2—diffusion in L2

In this case, it is considered that the convection also exists in L2 as a tracer flow mechanism in addition to before mentioned ones. In the discretization process, the boundary condition of tracer flow from the wellbore to its adjacent grid is different from other grids.

**Figure 4:**
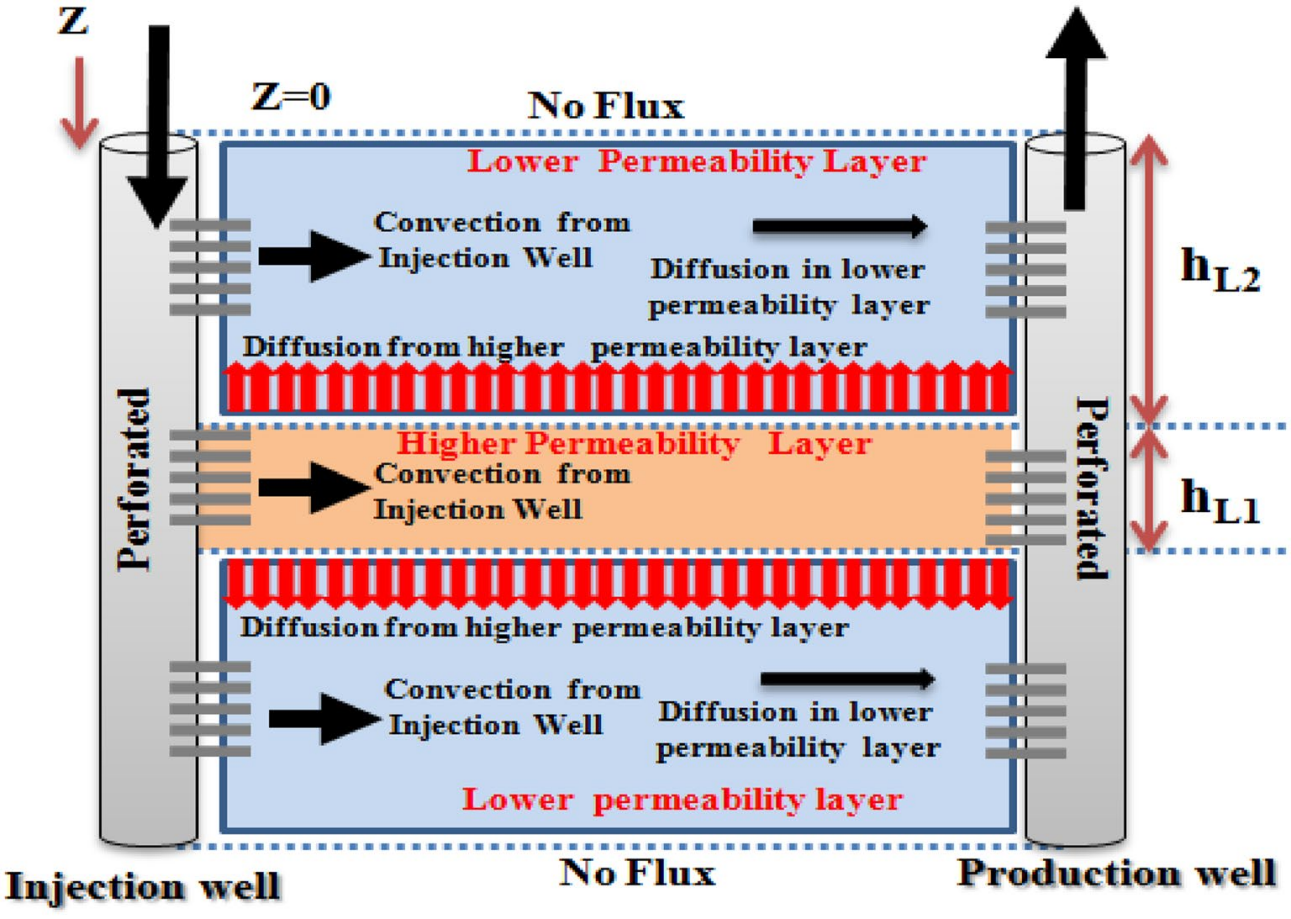
Schematic of convection–diffusion tracer mass transfer (convection in L1—convection in L2—diffusion from L1 to L2—diffusion in L2).

In this case, the rates of tracer flow from the wellbore toward the L1 and L2 will be different. Due to this difference, a new dimensionless Peclet number (P_em_) will be introduced to describe the convection flow of the tracer from the wellbore toward the lower permeability layer (L2). This case is similar to the dual permeability mechanism in fractured reservoirs. The initial and boundary conditions are similar to the previous case. The schematic of flow in case III is illustrated in Fig. 4.

The definition of the new Peclet number and also general convection–diffusion equation in L2 will be presented in the following forms:

46 $$P_{e2} = \frac{{\frac{{rq_{o} }}{{2\pi rh\phi_{2} }}}}{{D_{2} }} = \frac{{rV_{2} }}{{D_{2} }}$$

47 $$\frac{{\partial C_{2} }}{\partial t} = D_{2} \frac{{\partial^{2} C_{2} }}{{\partial r^{2} }} + \frac{{D_{2} }}{r}\left( {\frac{{\partial C_{2} }}{\partial r}} \right) + D_{2} \frac{{\partial^{2} C_{2} }}{{\partial Z^{2} }} - v_{2} \frac{{\partial C_{2} }}{\partial r}\quad {\text{(dimentionl)}}$$

48 $$\frac{{\partial C_{2D} }}{{\partial t_{D} }} = \frac{{\partial^{2} C_{2D} }}{{\partial r_{D}^{2} }} + \frac{1}{r}\frac{{\partial C_{2D} }}{{\partial r_{D} }} + \frac{{\partial^{2} C_{2D} }}{{\partial Z_{D}^{2} }} - (Pe_{2} /r_{D} )\frac{{\partial C_{2D} }}{{\partial r_{D} }}\quad {\text{(dimentionless)}}$$

To solve the mass transfer equation numerically in the three mentioned cases, some assumptions were considered in the modeling. The time of simulation (dimensionless time) is assumed to be 10^4^. The value of the porosity ratio (the porosity of L2 to L1 ratio) is considered to equal five. The value of time difference (dt) is assumed to be equal to 0.5, the value of horizontal changes (dz) equals to 0.5 and the value of the radial change (dr) equals to 5 in numerical modeling. Therock and fluid properties and also the porosity ratio of the low permeable to the high permeable layer is assumed to be constant; therefore, the difference in porosity ratio in different grids is ignored. On the other hand the properties of the grids are assumed to be constant with time, except for the changes in concentration.

The dimensions of the grids are assumed unchanged in numerical calculations. The possible existence of diffusion mechanism from the low permeable layer to the high permeable layer has been neglected. The other assumed parameters are presented in Table 2 which they may be changed for sensitivity analysis in the following section.

**Table 2:** Defined dimensionless parameters for numerical convection–diffusion calculations.

Parameter	Value
Dimensionless simulation time	10^4^
Dimensionless ratio o lower permeability layer thickness to wellbore radius (h_r_)	6
Porosity ratio ($\frac{{\phi_{2} }}{{\phi_{1} }}$)	5
Dimensionless dispersion coefficient (*α*_*D*_)	20
Dimensionless radius (*r*_*D*_)	200
Time difference (dt)	0.5
Difference in vertical direction (dz)	0.5
Difference in radial direction (dr)	5
Dimensionless diffusion coefficient in L2 (D_L2_)	10^−9^
Dimensionless diffusion coefficient in L1 (D_L1_)	10^−5^
Peclet number in L1	10^4^
Peclet number in L2	10
Number of grids in vertical direction for low permeable layer (N_low_k_)	10
Number of grids in radial direction for high permeable layer (N_high_k_)	20

On the other hand, other assumptions will be introduced individually in each case. The total number of discretization grids from the injection well to a production well is assumed to be 220 grids for L1 and L2 in which N low_k and N high_k are equal to 10 and 20 respectively according to Eq. (32). In our study, according to (r, z + 1) nomination in the schematic of Fig. 2B, the first grid number of the higher permeable layer will be described by grid (1,11) and the final grid of permeable layer will be shown by (20,11). In another word, the value of **r** changes from 1 to N high_k, and the value of **z** + 1 will change from 1 to N low_k + 1 in grid numbering.

The detailed procedure to solve the convection–diffusion problem is presented in Appendix B for a twelve (12) grid system in which (N low_k + 1 × N high_k) equals 12 grids according to Fig. 2.

## Results and discussion

In this section, the result of convection–diffusion equations in the above-mentioned cases will be discussed. First of all, a comparison between analytical and numerical calculations will be evaluated to check the accuracy of numerical modeling. Then sensitivity analysis on affected parameters in the concentration profile and also the comparison between cases will be investigated in more details.

### Comparison between analytical and numerical modeling of tracer flow in a multilayered reservoir

Although analytical solutions are generally considered to be more rigorous than numerical methods due to providing exact solutions, sometimes these methods might not be able to handle complex problems with specific assumptions. As it mentioned in previous section, three different cases with different boundary condition will be discussed in this study in which only the first case is solved using analytical methods in literature^25^.

It is obvious that in order to compare the numerical and analytical solutions, the values of $\frac{{\phi_{2} }}{{\phi_{1} }}$, hr, αD, rD, and also simulation time are assumed to be equal in both solution methods.

The analytical solution of a mass transfer equation is solved in base case (Eqs. 29 and 30) which is simple case, using the following formula in Laplace (S) domain^25^:

49 $$\frac{{{\text{d}}^{2} {\overline{\text{c}}}_{{{\text{D}}2}} }}{{{\text{dz}}_{{\text{D}}}^{2} }} = {{\text{s}}\overline{\text{c}}}_{{{\text{D}}2}} - 0$$

50 $$\frac{{{\text{d}}^{2} {\overline{\text{c}}}_{{{\text{DL}}1}} }}{{{\text{dr}}_{{\text{D}}}^{2} }} - \frac{1}{{{\upalpha }_{{\text{D}}} }}\frac{{{{\text{d}}\overline{\text{c}}}_{{{\text{DL}}1}} }}{{{\text{dr}}_{{\text{D}}} }} - {\text{f}}\left( {\text{s}} \right){\text{r}}_{{\text{D}}} {\overline{\text{c}}}_{{{\text{DL}}1}} = 0$$

In which the ${\overline{\text{c}}}_{{{\text{D1}}}}$ and ${\overline{\text{c}}}_{{{\text{D2}}}}$ represent the concentration values of higher and lower permeability layers in Laplace domain respectively. The above equations can be solved by introducing the Ai as Airy function.

51 $${\overline{\text{c}}}_{{{\text{D}}2}} = \frac{{{\text{cosh}}\left( {\sqrt{\text{s}}\,{\text{Z}}_{{\text{D}}} } \right)}}{{{\text{cosh}}\left( {\frac{{\sqrt{\text{s}}\,{\text{h}}_{{\text{R}}} }}{2}} \right)}}{\overline{\text{c}}}_{{{\text{D}}1}}$$

52 $${\overline{\text{c}}}_{{{\text{D}}1}} = \frac{1}{{\text{s}}}{\text{e}}^{{\frac{{\left( {{\text{r}}_{{\text{D}}} - 1} \right)}}{{2{\upalpha }_{{\text{D}}} }}}} \frac{{{\text{Ai}}\left\{ {{\text{f}}\left( {\text{s}} \right)^{{{\raise0.7ex\hbox{${ - 2}$} \!\mathord{\left/ {\vphantom {{ - 2} 3}}\right.\kern-0pt} \!\lower0.7ex\hbox{$3$}}}} {*}\left[ {\frac{1}{{4{\upalpha }_{{\text{D}}}^{2} }} + {\text{f}}\left( {\text{s}} \right){\text{r}}_{{\text{D}}} } \right]} \right\}}}{{{\text{Ai}}\left\{ {{\text{f}}\left( {\text{s}} \right)^{{{\raise0.7ex\hbox{${ - 2}$} \!\mathord{\left/ {\vphantom {{ - 2} 3}}\right.\kern-0pt} \!\lower0.7ex\hbox{$3$}}}} {*}\left[ {\frac{1}{{4{\upalpha }_{{\text{D}}}^{2} }} + {\text{f}}\left( {\text{s}} \right)} \right]} \right\}}}$$

53 $${\text{f(s)}} = \frac{1}{{{\upalpha }_{{\text{D}}} {\text{*Pe}}}}\left[ {{\text{s}} + \frac{{{\uptheta }_{2} }}{{{\uptheta }_{1} }}\left[ {\frac{{2\sqrt{\text{s}}}}\,{{{\text{h}}_{{\text{R}}} }}{\text{tanh}}\left( {\frac{{\sqrt{\text{s}}\,{\text{h}}_{{\text{R}}} }}{2}} \right)} \right]} \right]$$

Finally the above equations will be inversed in time domain using the Stehfest algorithm.

**Figure 5:**
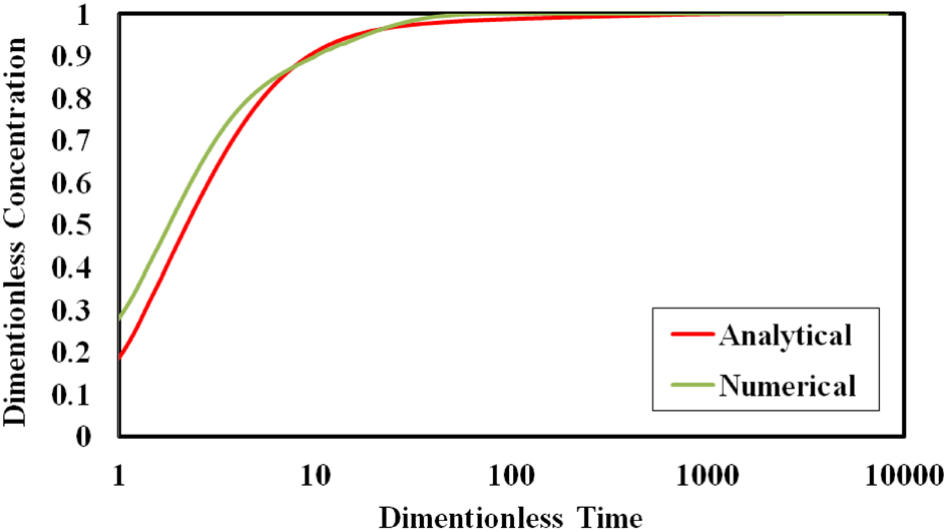
Comparison between concentration profile in analytical and numerical methods in semilog scale (Case1: convection in L1—diffusion from L1 to L2).

Due to complexity of assumptions in other cases, in this study, the numerical methods are selected to use for the investigation and evaluation of mass transfer equations in the three mentioned cases, but due to check the accuracy, the first case (Convection in L1—diffusion from L1 to L2) is solved by both the analytical and numerical methods. Figure 5 shows the comparison of the result where the average difference between the analytical and numerical values of calculated concentration is about 0.13%. Also, the maximum difference between the two methods is about 9.4% which shows good agreement and a reasonable range of difference.

### Numerical investigation of convection–diffusion mechanisms

#### Case I: (Convection in L1—diffusion from L1 to L2)

**Figure 6:**
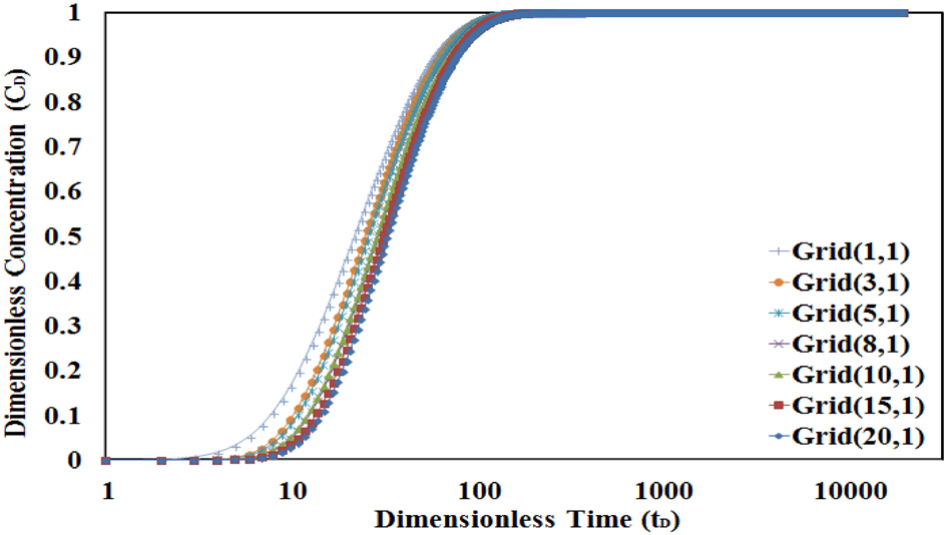
The concentration–time profile for L1. Grid numbering according to (r,z) gridding. Total number of grid in this condition is calculated according to Eq. (32).

Figure 6 shows the dimensionless concentration–time profile after numerical solving the mass transfer equation in the higher permeable layer (L1) regarding the convection mechanism. According to this figure, the value of concentration changes from its initial value (i.e. zero) to the final value (i.e. one) in different discretization grids. The figure shows that in the first grid (near the wellbore) the concentration rapidly yields the final value while its value in other grids gradually increases. It can be said that in a special time, the more the distance from the wellbore, the lower the concentration value of discredited grids will yield. The concentration value in all grids becomes unity sometimes later which these times are sensitive to affected parameters in the convection–diffusion equation.

**Figure 7:**
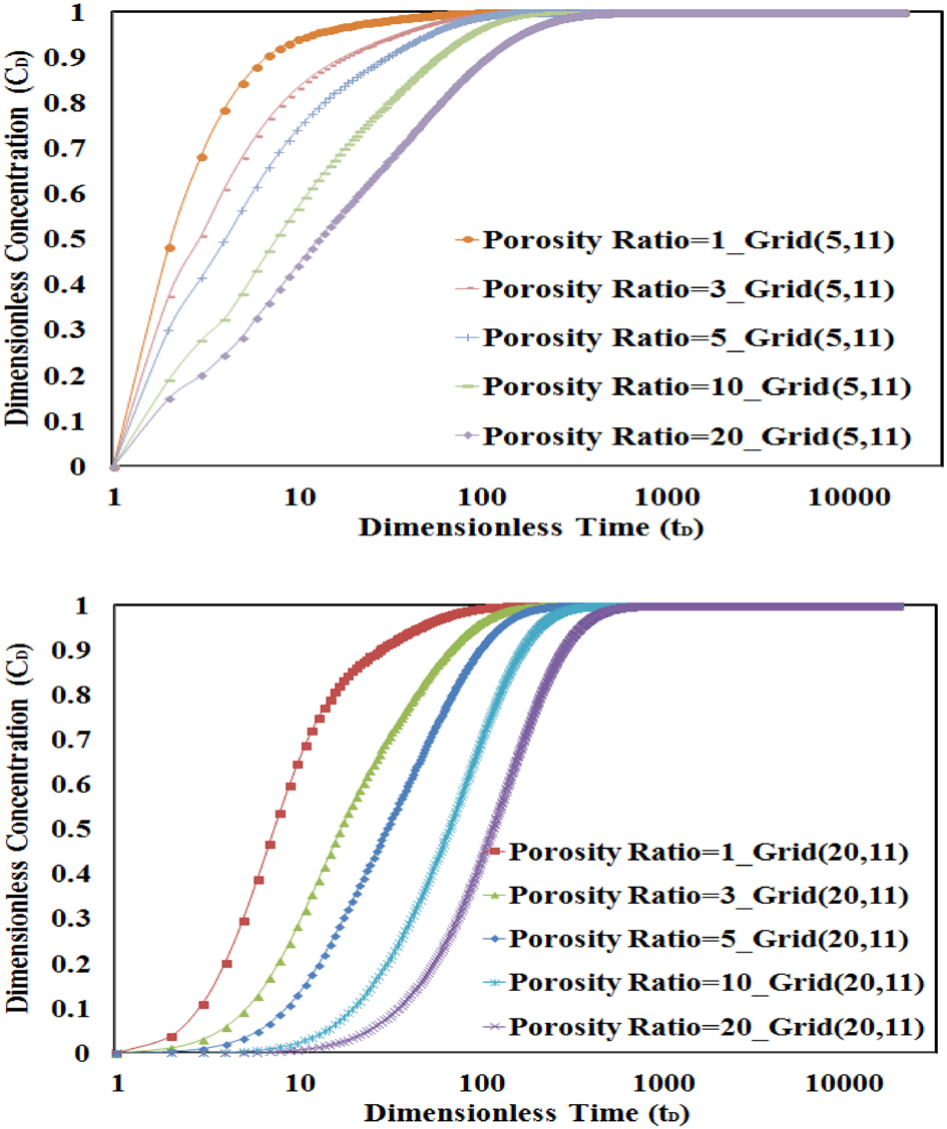
Sensitivity analysis of porosity ratio in L1 for numerical grids 5 and 20. The higher the porosity ratio, the more time is needed to get final concentration value.

As it is shown in Fig. 7, the higher the porosity ratio, the more time is needed to get the final concentration value. It can be said that in cases of higher porosity ratio, due to higher pore volume in L2, it will need more time to yield the final value of concentration in L1 and L2.

Also, this figure shows that the time to reach the final value of concentration in L1 will be increased in higher numbers of the discretized grid for all values of porosity ratio. As an example, it is shown that the concentration profile will yield faster to the final value in the numerical grid (5, 11) in comparison to the numerical grid (20, 11) because of the lower distance to the source of tracer injection.

**Figure 8:**
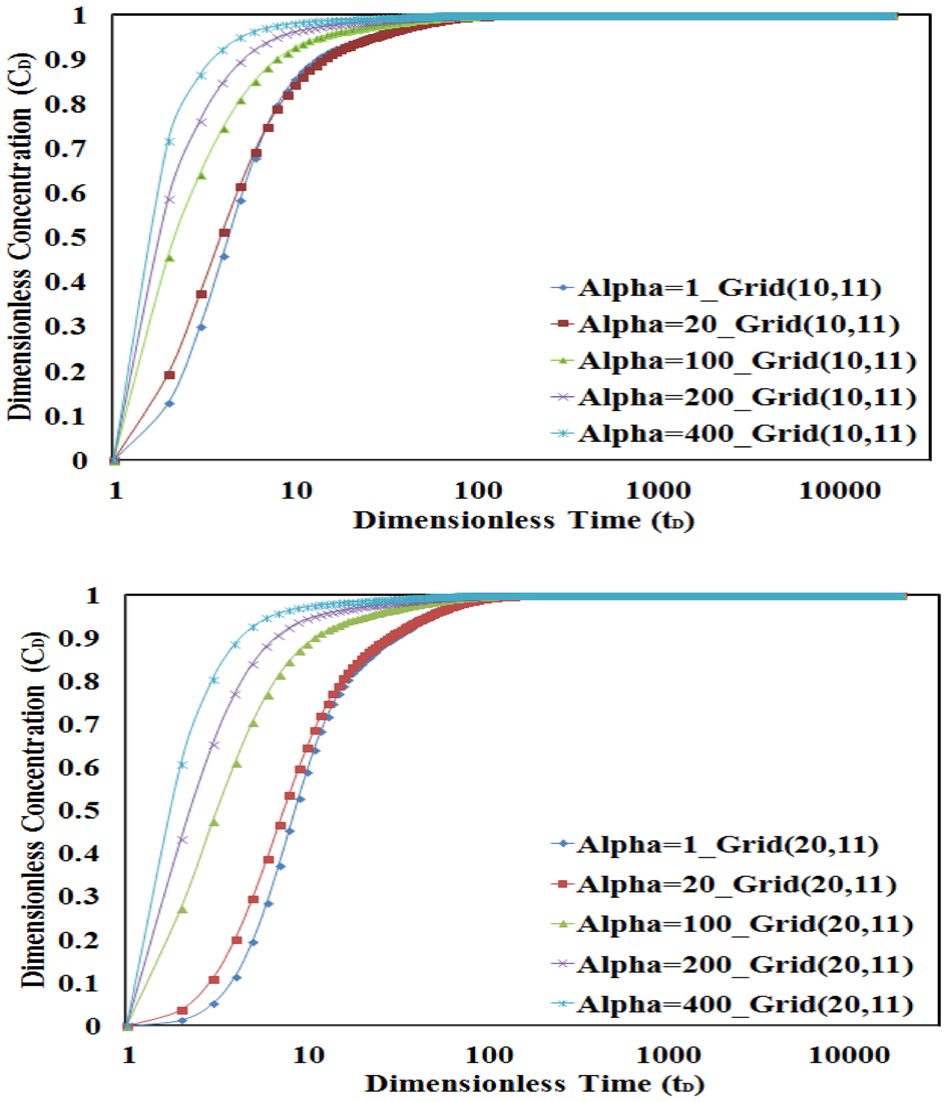
Sensitivity analysis of the dispersivity in L1 for numerical grids 10 and 20. The sharp and fast concentration increase in lower times will be seen in higher dispersivity values.

On the other hand, Fig. 8 shows that by increasing the value of the dispersivity coefficient, the time needed to gradually increase the concentration will decrease i.e. the higher the dispersivity coefficient, the sharp and fast increase in concentration occurs in lower times.

**Figure 9:**
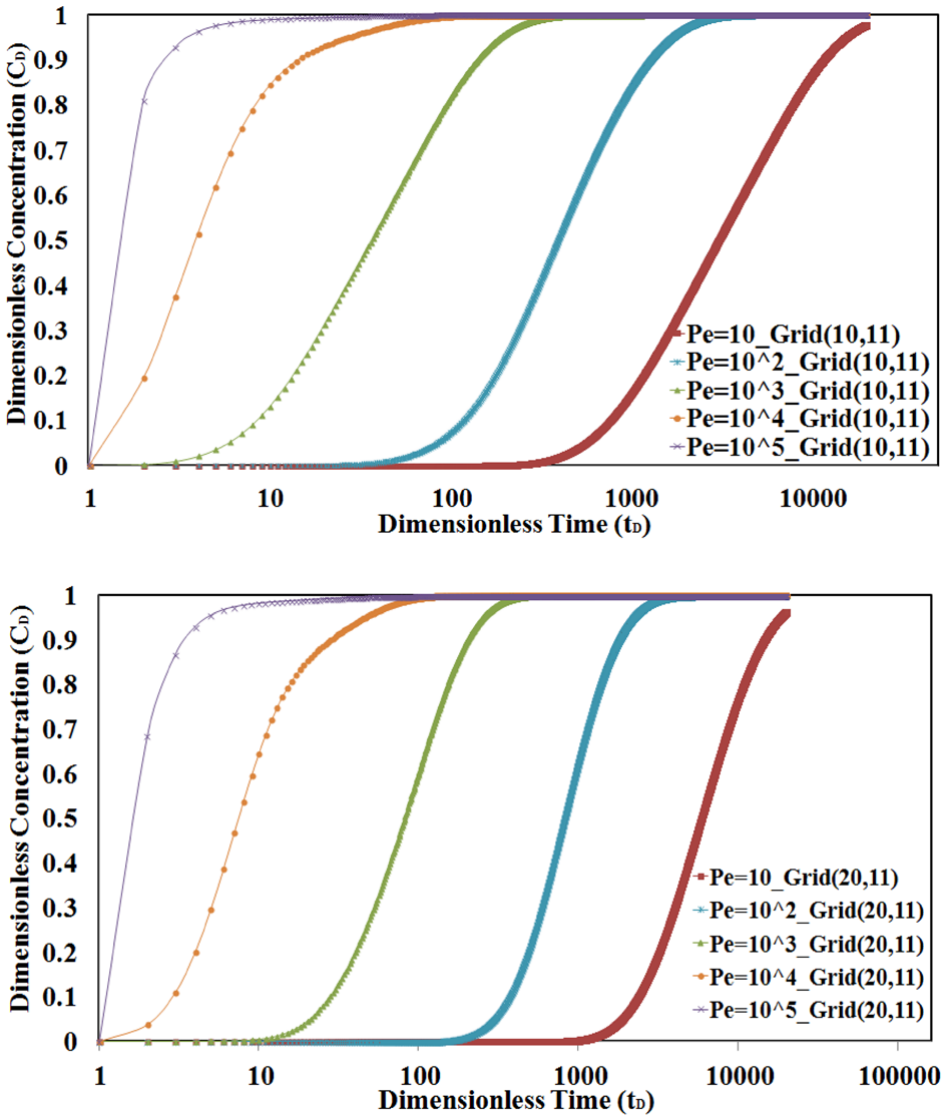
Sensitivity analysis of Peclet number values in L1 for numerical grids 10 and 20. The convection mechanism is dominant in higher Peclet values.

The value of the Peclet number is another important affected parameter in concentration–time profile evaluation. By increasing the value of the Peclet number, the concentration will be faster received to its final value. Also due to less effect of the convection mechanism in lower values of Peclet number, the time to reach the final concentration value is increased in low Peclet numbers. This behavior is depicted in Fig. 9.

**Figure 10:**
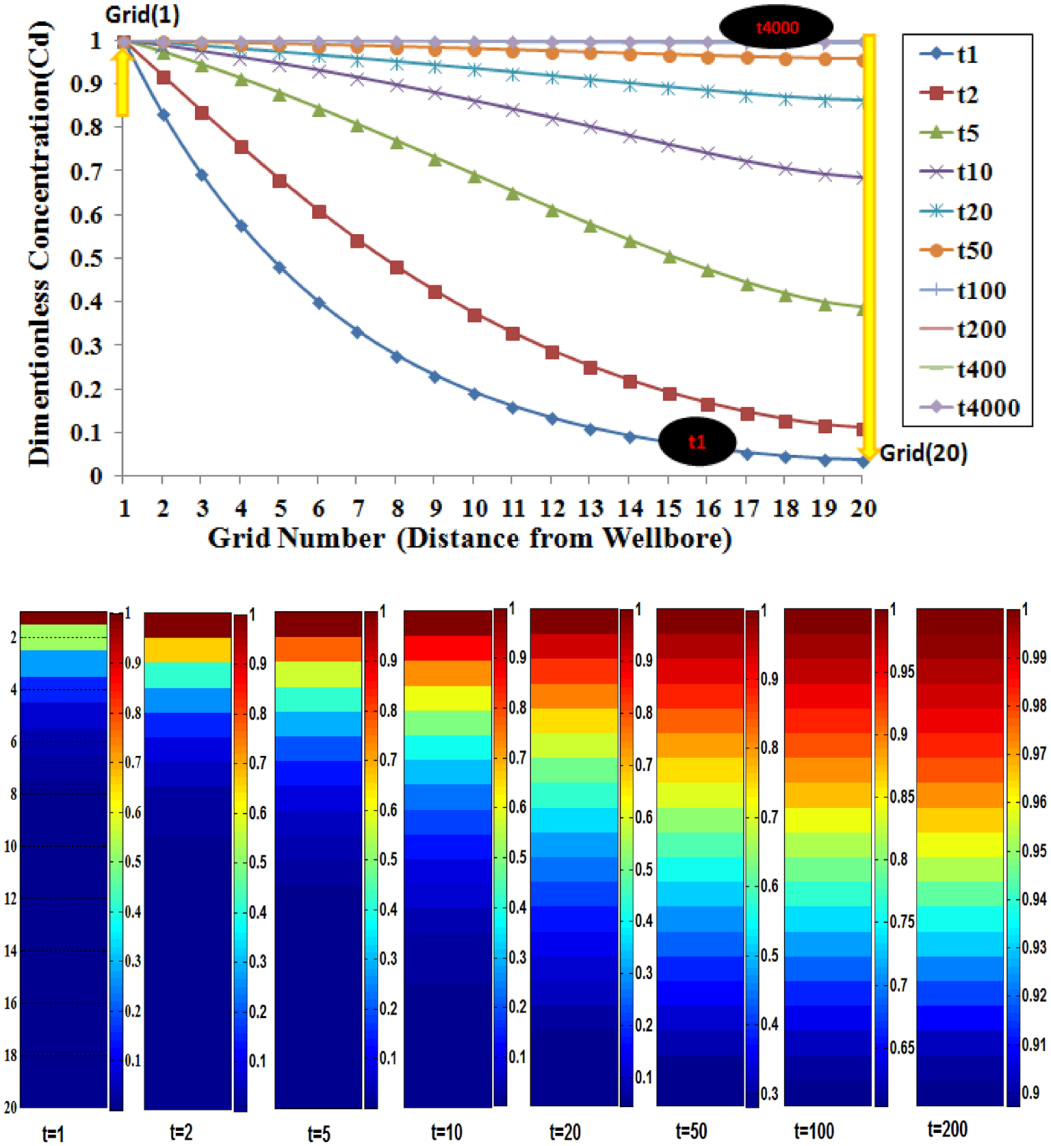
Concentration-distance profile for L1 in different times. The more the time consuming, the higher the value of concentration in special location will be occurs to meet final concentration value.

The variation of concentration versus location at different times for the first layer also is shown in Fig. 10. According to the curves illustrated in this figure, for a special location in the radial direction of tracer flow (discretized grids), the value of concentration will change at different dimensionless times. It can be said that the more time consume, the higher the value of concentration in a special location will occur to meet the final concentration value.

After discussing the concentration variation in L1, the profile of concentration time in L2 will be evaluated in the following figures. In this condition, only diffusion from L1 to L2 is included in calculations for this layer.

**Figure 11:**
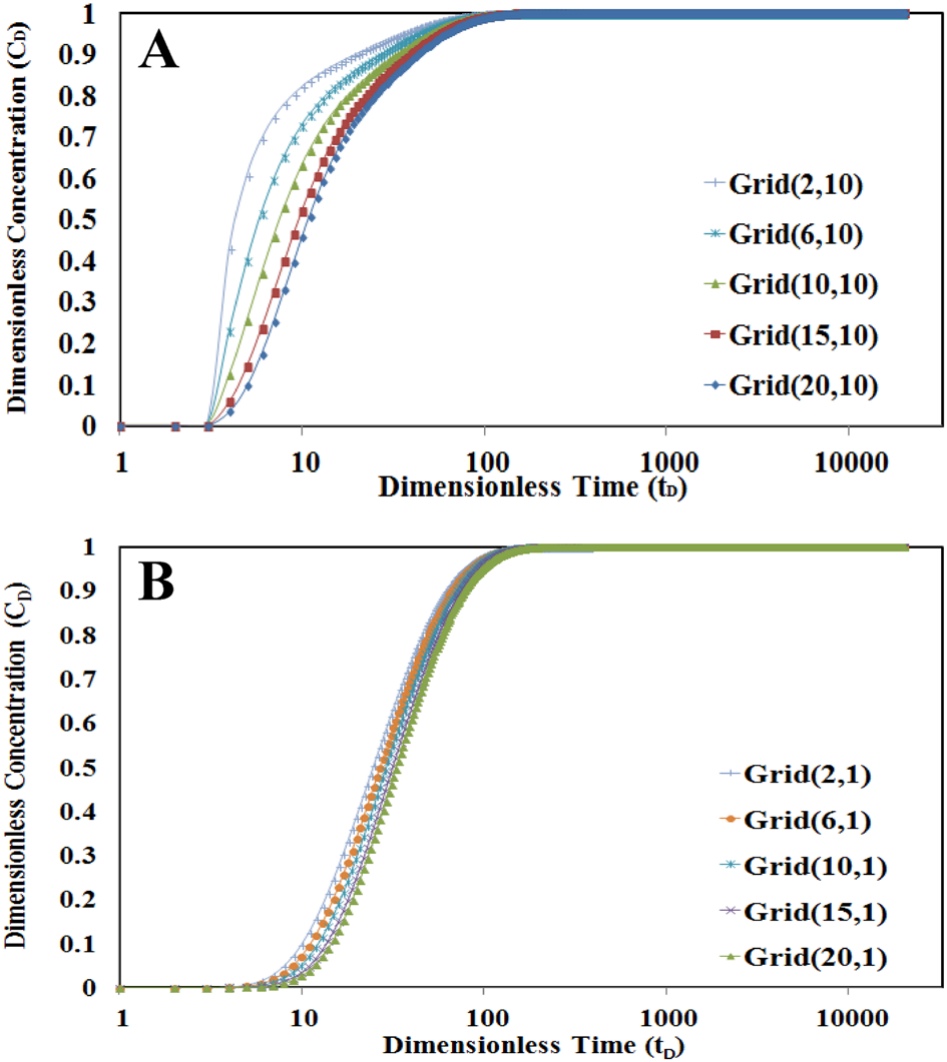
The concentration–time profile for L2, (**A**) grids near the L1, (**B**) grids far from L1. Grid numbering according to (r,z) gridding.

As is shown, the shape of the concentration profile in grids near layer L1 (Fig. 11a) is different in comparison to the grids far from this layer (Fig. 11b). It is also obvious that the trend of increasing concentration is faster in grids near L1 than in the grids for this layer.

Although the trend of concentration value approaches to unity in L2 like L1, because of the diffusion mechanism and also lower permeability values, the final concentration will be yielded at later times.

#### Case-II: (convection in L1—diffusion from L1 to L2—diffusion in L2)

In this case, the diffusion mechanism in L2 will be included in addition to diffusion from L1 to L2. Therefore two new boundary conditions will be used in numerical calculations of mass transfer for this layer. The concentration profile for the higher permeability layer (L1) is similar to the previous case.

**Figure 12:**
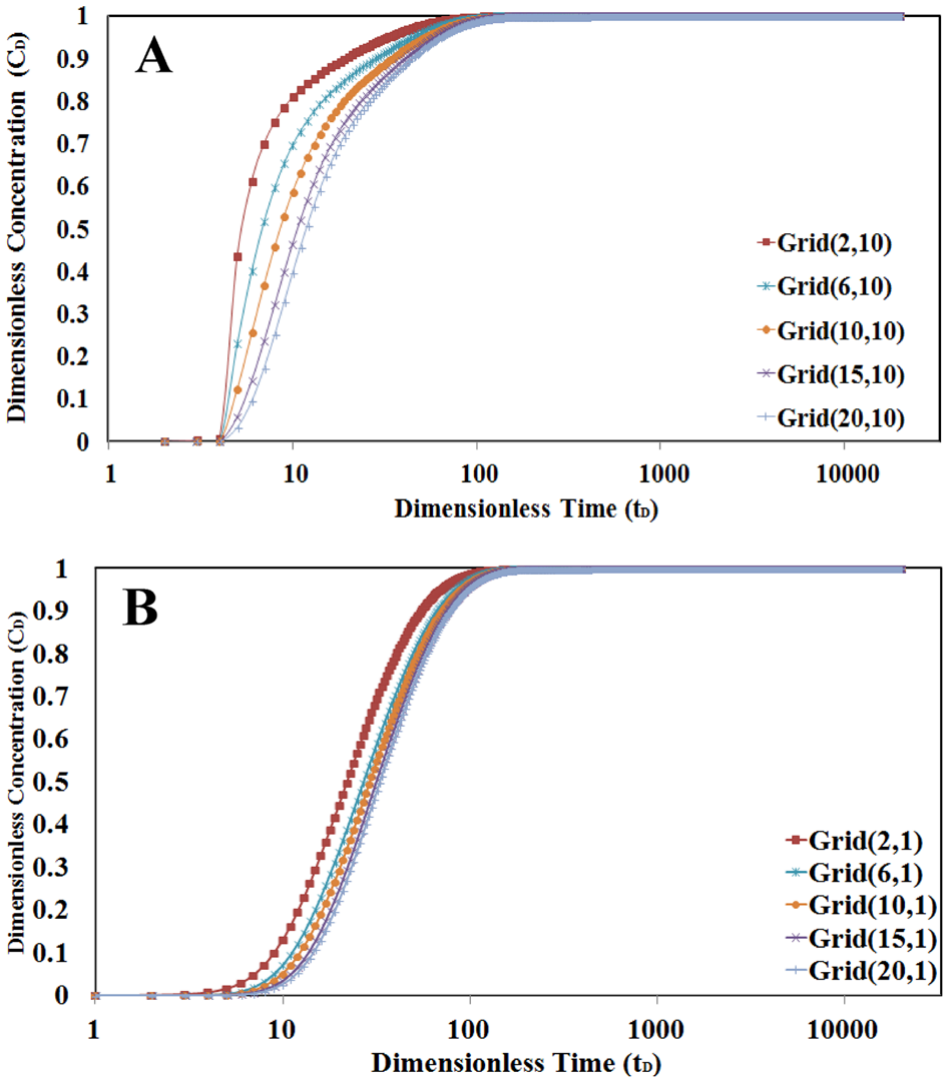
The concentration–time profile for L2 (**A**) Grids near L1 (**B**) grids far from L1. Grid numbering according to (r,z) gridding.

In this case, adding a new diffusion mechanism may not have so significant effect on mass transfer calculation. It is because, in higher values of the Peclet number, the convection mechanism is dominant. In this condition, the concentration–time profile wouldn’t be so different in comparison to case 1 as shown in Fig. 12.

To better understand the importance of the diffusion mechanism, especially in L2, a sensitivity analysis is done on Peclet number values. To compare the results, two grid numbers are selected and the concentration–time profile will be evaluated for different Peclet numbers in cases I and II. To do this, the grid number (10, 1) will be selected. Because the effect of diffusion is so important in low Peclet number values, the values of 10, 100, and 1000 for this dimensionless number were considered for comparison.

**Figure 13:**
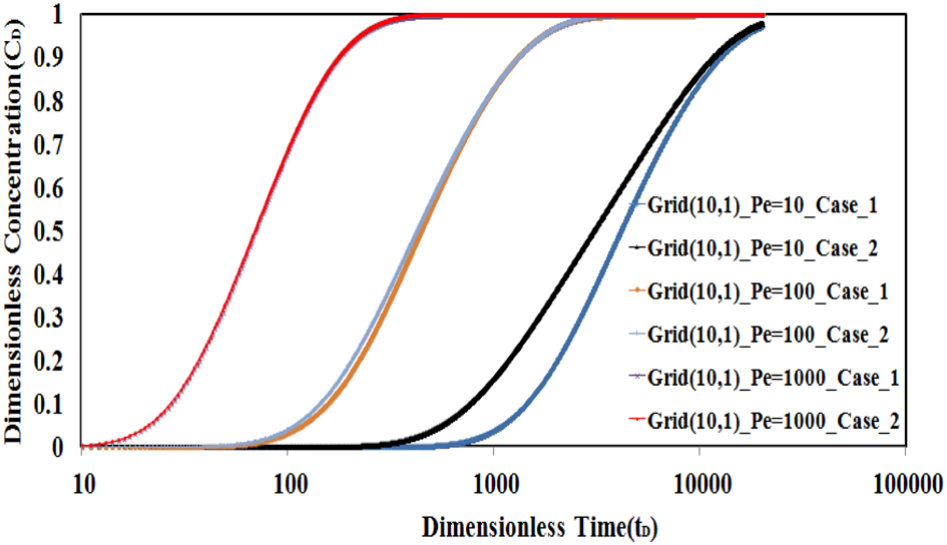
The comparison between profiles of concentration in cases I and II. The difference between concentration profiles in two cases is considerable in low Peclet numbers.

A comparison of the results of the concentration–time profile in Case I and Case II is shown in Fig. 13. According to mentioned figure, the difference between concentration profiles in the two cases is considerable in a low Peclet number of 10 where the diffusion mechanism is dominant. This behavior is more common in low permeable mediums such as single/multilayered tight or shale reservoirs.

The above figure also shows that the effect of tracer diffusion in L2 is not considered when values of the Peclet number increase in the range of convection dominance.

#### Case-3: Convection in L1—convection in L2—diffusion from L1 to L2—diffusion in L2

In this case, the convection mechanism in L2 is also included in addition to other defined mechanisms in previous cases. Although the concentration–time profile for L1 is not the case of change, due to including the convection mechanism in calculations of L2, the concentration profile will be affected by a considerable change in comparison to the other cases. The results of this case can simulate the convection–diffusion flow mechanisms in the real porous medium.

**Figure 14:**
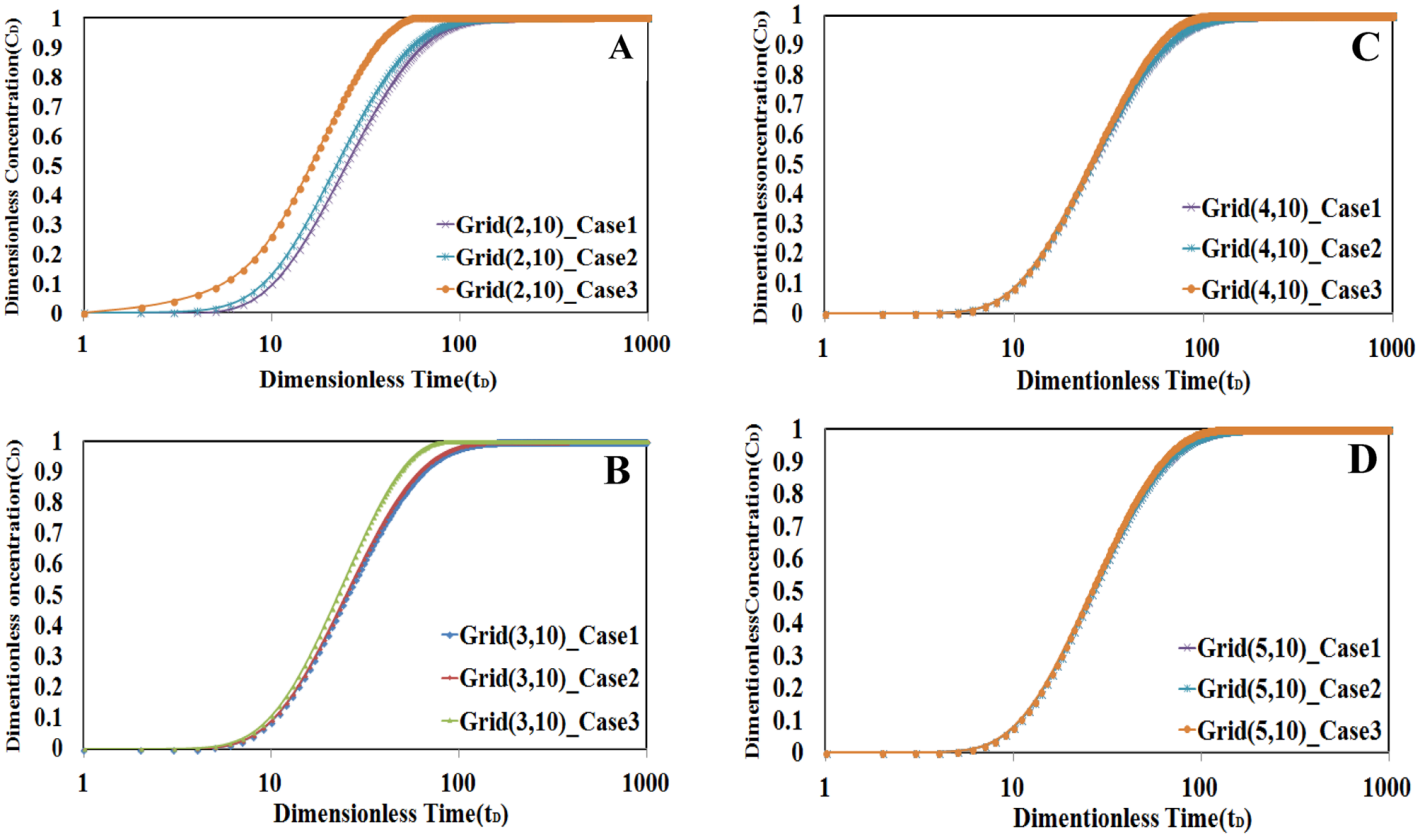
The comparison of concentration–time profile for cases I, II and III. The difference in profile near the injection well (**A**, **B**) is higher than this profile in locations farther from injection point (**C**, **D**).

According to Eq. (44), a new Peclet number will introduce and used to include the convection in L2. In this condition, the value of the Peclet number for L2 will be selected as equal to 100. Figure 14 shows the comparison of the concentration–time profile for cases I, II, and III. The difference in profile near the injection well (tracer source) is significant in case 3 compared to the two before-mentioned cases (Fig. 14A,B), but the difference becomes less in the more distant locations (Fig. 14C,D). It can be said that the effect of including convection in the low permeable layer is considerable at initial locations, but the effect of diffusion from higher to lower permeable layer will be more important and affect the results in farther locations.

## Conclusions

In the current study, a detailed numerical evaluation of the convection–diffusion equation in layered reservoirs is discussed using tracer response. The dispersivity coefficient, the effect of some related parameters to Peclet number, as well as changes in the concentration profile of the tracer, especially at low Peclet numbers, have also been investigated. On the other hand, the time of importance and dominance of Convection and Diffusion mechanisms in the low permeable layer is also one of the main subjects that was evaluated in this study. The main results of this paper are summarized as follows:

- The diffusion mechanism is dominant and has a more visible effect in low permeable mediums such as single/multilayered tight or shale reservoirs. Also, diffusion mechanism is more considerable in lower values of Peclet number.
- As the porosity ratio of low-to-high permeability layer increases, more time is needed to reach the final concentration value. Also by increasing the dispersivity coefficient, the sharp and fast increase in concentration occurs in smaller times.
- Although the trend of concentration in the low and high permeable layers are similar to each other, because of diffusion mechanism and also permeability values, the maximum concentration in low permeable layer will yield at later times.
- The effect of including convection in the low permeable layer is considerable at initial locations near the tracer injection point, but the effect of diffusion from the higher to lower permeable layer will be more important and affect the results in farther locations.

### Supplementary Information

Supplementary Information.

## Data Availability

The data used during the current study are available upon reasonable request.

## Abbreviations

C: Solute concentration (M/L^3^)
D_d_: Diffusion coefficient (L^2^/T)
D_L_: The longitudinal hydrodynamic dispersion (L^2^/T)
D_T_: The transverse hydrodynamic dispersion (L^2^/T)
D_2_: Dimensionless diffusion coefficient in L2
dA: Cross sectional area of element
dz: Difference in vertical direction
dr: Difference in radial direction
$$\frac{{{\text{dC}}}}{{{\text{dx}}}}$$: Concentration gradient (M/L^3^/L)
$\frac{{{\text{dC}}}}{{{\text{dt}}}}$: Change in concentration with time (M/L^3^/T)
F: Mass flux of solute per unit area per unit time (M/L^2^T)
F_i_: One dimentional mass flux in i direction
i: Direction normal to cross sectional area
n_e_: Effective porosity
N_*low-k*_: Number of grid in L2
N_*high-k*_: Number of grid in L1
r: Radial distance of the well
R: Length of well screen or open bore hole
V: Average pore velocity of injection
*α*_*L*_: Longitudinal dispersity (m)
*α*_*T*_: Transverse dispersity (m)
*α*_*D*_: Dimensionless dispersion coefficient
$\frac{{\phi_{2} }}{{\phi_{1} }}$: Porosity ratio of low to high permeable layers
