# Supplementary material for: Detailed numerical evaluation of diffusion convection equation in layered reservoirs during tracer injection
Source: Sci Rep. 2023 Sep 11;13:14989. doi: 10.1038/s41598-023-40934-8 (PMC10495337; doi:10.1038/s41598-023-40934-8)
Supplement: Supplementary file 1 — Supplementary Information. [file 41598_2023_40934_MOESM1_ESM.docx]

**Appendix-A**

The mass transfer equation in the high permeable layer will be derived in the following forms. First of all, it is assumed that diffusion is included in calculations. The diffusion term from the higher to lower permeability layer is also included as the source term.

If the diffusion coefficient term is equal to zero, then the equations will be presented in the following form:

**Appendix-B**

To solve the convection-diffusion equation by numerical methods, the equations related to space will be denoted by first and second-order derivatives and they will be written in central and foreword forms respectively. Also, the equation related to time will be written in central derivative form.

Also, the number of rows and columns in the matrix of coefficients will be calculated according to the following equation:

(N__L_ +1)×(N__H_) = NN (B-1)

In which N_L describes the number of grids in the lower permeable layer and N_H indicates the number of grids in the higher permeable layer. The number of values in known and unknown matrixes for numerical calculation equals (NN*1) and the number of coefficients matrix values in numerical gridding equals (NN*NN).

As an example for the numerical solution of the convection-diffusion equation, a 3x3 grid system of low and high permeable layers with a total number or row and column in coefficients matrix equal to 12 (4x3) will be defined for calculation. It should be noted that more numbers of discretization (gridding) can be used to solve the mass transfer equation in the method mentioned in the following sections.

According to Figure 2 in this study and using predefined initial and boundary conditions, the discretization for the first grid is in the following form where diffusion from higher to lower permeable layer is included:

The expressions on the left-hand side of equation B-5 are one of the known matrixes cells in current time (equals n). The remnant expressions in the right-hand side of this equation explain the matrix coefficient cells and the concentrations in future time (n+1) denoted the unknown matrix cells.

The discretization of other grids for the lower permeable layer is like the first grid regarding their specific boundary conditions. On the other hand discretization in one grid of the higher permeable layer including the convection mechanism is in the following form:

In the above discretization of the higher permeable layer, equations B-9 up to B-12 are the expressions for the matrix of coefficient, and equation B-13 describes the known matrix values.

After defining the known and coefficient matrixes using the discretization of high and low permeable layers, the convection-diffusion equation can be solved numerically using the AX=B where A, X, and B express the coefficient, unknown, and known matrixes respectively.

**Appendix-C**

In this appendix, a brief history of activities performed in the analysis of tracers is presented.

| Year | Author | Type of  Investigation | Description | No |
| --- | --- | --- | --- | --- |
| 1968 | Deem | Case Study | The first tracer test to measure S_or_ in East Texas field | 33 |
| 1973 | Tomich | Modeling or simulation | Using the idea presented by Deans to measure S_or_ in a single well tracer test | 34 |
| 1974 | Deans - Shallenberger | Case Study | The first application of a single well tracer test to measure S_wi_ using an idea developed by Deans. The greater the difference in separation between the two tracers, the greater the saturated water saturation | 35 |
| 1980 | Deans, H.A. and Majoros | Case Study | Presenting a report on the theory and operational design of single well tracers. | 36 |
| 1986 | Carlisle-Deans | Modeling or simulation | Present a new model to investigate the tracer flow in carbonated formations | 37 |
| 1987 | Ohno | Case Study | Using interwell tracer analysis to evaluate the heterogeneity in a heavy oil field | 38 |
| 1989 | Seetharam | Modeling or simulation | Investigation the effect of interlayer flow on the motion of the tracer and its test interpretation | 39 |
| 1991 | Tang | Laboratory  /field application | Using radioactive tracers to measure the value of S_o_ in laboratory and field case of gas injection | 40 |
| 1991 | Park et al. | Modeling or simulation | Evaluation of Temperature effect in tracer injection | 41 |
| 1992 | Portel et al. | Modeling or simulation | Using the convection-dispersion equation to describe tracer flow in homogenous formations and dead end and porous sphere models in heterogeneous formations | 42 |
| 1994 | Yi et al. | Modeling or simulation | Present a numerical method to modeling the tracer flow in multi well tracer test of heterogeneous formations | 43 |
| 1995 | Datta guppa | Modeling or simulation | Generate type curves using defined parameters in tracer flow tests | 44 |
| 1996 | Loula et al. | Modeling or simulation | Present a method to simulate tracer flow using finite element method | 45 |
| 1996 | almeida | Modeling or simulation | Using new integral methods to analytically solve the two-dimensional equations of the tracer in steady and single-phase flow in the five spot water injection model | 46 |
| 1996 | Abbaszadeh | Modeling or simulation | Using tracers to investigate the performance of thin layers participating in flow | 47 |
| 1997 | Dattagupta | Modeling or simulation | Comparison the accuracy of permeability estimation based on tracer analysis and transient pressure analysis and suggesting the use of both tests to increase the accuracy of permeability estimation. | 48 |
| 1997 | Khaled Aziz | Modeling or simulation | Investigating the reduction of numerical errors by simulating and solving tracer equations with the special finite difference method. | 49 |
| 1998 | ghori | Other Topics | Quantitative investigation of permeability heterogeneity using interwell tracer test analysis. | 50 |
| 2000 | Tang | Modeling or simulation | Investigating the measurement of S_or_ in the presence of mobile oil using tracer tests. | 51 |
| 2001 | Tang | Case Study | investigating S_or_ in one of the carbonate fields using single-well and inter-well tracer tests | 52 |
| 2003 | Mahadevan et al | Case Study | Investigating the category of dispersion and its coefficient in porous media and introducing 3 different types of dispersion in field conditions. | 53 |
| 2003 | Guevarajordan | Modeling or simulation | The use of new numerical methods such as finite element, to numerically solve the tracer equations in heterogeneous reservoirs. | 54 |
| 2003 | Kocabas | Modeling or simulation | Presenting an analytical model to investigate tracer flow in oil reservoirs with high permeability medium | 55 |
| 2005 | Samaniego | Modeling or simulation | Presenting new models to investigate tracer flow in homogeneous and fractured reservoirs by applying dimensionless groups similar to well testing and estimating reservoir parameters | 56 |
| 2007 | Li et al. | Modeling or simulation | Investigating three-dimensional modeling of tracer flow to determine gas trapping in foam injection. | 57 |
| 2011 | Huseby | Modeling or simulation | Presenting a new method of tracer simulation with a higher speed than usual simulations and evaluation of gas tracer tests to investigate gas injection performance. | 58 |
| 2012 | Cheng | Modeling or simulation | Using the interwell tracer test to check the amount of injectivity and sweep efficiency of surfactant injection before conducting field test. | 59 |
| 2013 | Kocabas | Modeling or simulation | Presenting an analytical and numerical model to study tracer flow in a reservoir with high permeability layers and evaluation by Comsol numerical simulator | 60 |
| 2015 | Arief | Modeling or simulation | Presenting a method using tracer data to obtain more accurate results in re-injection process of the production water in reservoir | 61 |
| 2016 | Dean | Modeling or simulation | The use of tracer during polymer injection and estimation of S_or_ after polymer injection in heterogeneous reservoirs | 62 |
| 2016 | al-shalabi | Modeling or simulation | Modeling and simulation of single-well tracer test of water injection with low salinity in a carbonate reservoir using UTICHEM simulation software in non-isothermal, three dimensional, multi-phase and multi-component conditions and determination of residual oil saturation | 63 |
| 2017 | Shen | Modeling or simulation | Evaluating the presence of cross flow in reservoir using tracer analysis | 64 |
| 2018 | Alabbad | Case Study | Introducing the new composition of single-well tracer and investigating the small volume needed to perform field-scale testing and examining the response of the results. | 65 |
| 2021 | [Davidescu](javascript:;)  et al | Case Study | Assessment of Sweep Efficiency in a Multi-Layered Reservoir Based on Consecutive Inter-Well Tracer Tests | 66 |
